# Authors' Reply

**DOI:** 10.1371/journal.pmed.0030116

**Published:** 2006-02-28

**Authors:** Jeffrey R Lacasse, Jonathan Leo

**Affiliations:** **1**Florida State UniversityTallahassee, FloridaUnited States of America; **2**Lake Erie College of Osteopathic MedicineBradenton, FloridaUnited States of America

We are pleased to see that our Essay [ 1] has sparked this discussion regarding consumer advertising of psychiatric medications.

There seems to be no disagreement with our main theses—antidepressant advertisements do not accurately represent the evidence base from psychopharmacology, experimental psychiatry, and neuroscience; are not strictly based on the United States Food and Drug Administration (FDA)-approved prescribing label; and may mislead consumers.

We believe many have bought into the serotonergic hypothesis of depression/generalized anxiety/social anxiety/obsessive-compulsive disorder/panic disorder/post-traumatic stress/bulimia/premenstrual dysphoric disorder largely because the serotonin reuptake inhibitor (SSRI) medications are licensed for these conditions. We reemphasize that pathophysiology cannot be established through clinical efficacy [ 2], yet this critical point seems to have been largely overlooked, particularly by regulators.

At the date of this letter, the advertising we presented in our Essay is still widespread, and quite visible on consumer advertising Web sites of SSRI manufacturers.
